# Arthroscopic release for frozen shoulder: Does the timing of intervention and diabetes affect outcome?

**DOI:** 10.1371/journal.pone.0224986

**Published:** 2019-11-11

**Authors:** Yu-De Su, Tien-Ching Lee, Yu-Chuan Lin, Shen-Kai Chen

**Affiliations:** 1 Department of Orthopaedics, Kaohsiung Medical University Hospital, Kaohsiung Medical University, Kaohsiung, Taiwan; 2 Orthopaedic Research Center, College of Medicine, Kaohsiung Medical University Hospital, Kaohsiung Medical University, Kaohsiung, Taiwan; 3 Graduate Institute of Medicine, College of Medicine, Kaohsiung Medical University Hospital, Kaohsiung Medical University, Kaohsiung, Taiwan; 4 Department of Orthopedics, Kaohsiung Municipal Ta-Tung Hospital, Kaohsiung Medical University Hospital, Kaohsiung Medical University, Kaohsiung, Taiwan; Universidade Federal Fluminense, BRAZIL

## Abstract

**Purpose:**

To evaluate the effect of timing of arthroscopic release and manipulation under anesthesia for frozen shoulder in patients with diabetes and non-diabetes.

**Methods:**

One hundred and twenty-seven patients with frozen shoulder were included in the study. Each patient was assigned to: 1) one of four groups according to the duration from symptom onset to surgery (group A: ≤3 months; group B: 3–6 months; group C: 6–12 months; group D: >12 months), 2) diabetic or nondiabetic group. The outcomes were measured by shoulder range of motion (ROM), Disabilities of the Arm, Shoulder, and Hand (DASH) score, American Shoulder and Elbow Surgeons (ASES) Shoulder score, the period of pain relief, overall duration of disease, and satisfaction.

**Results:**

All the patients got great improvement in shoulder ROM (P < .0001) after arthroscopic surgery, but there was no statistical difference in the pre-operative and post-operative shoulder ROM between the four groups and between diabetic and nondiabetic groups. The overall duration of disease was mean 55.4~68.7 weeks, which demonstrated much shorter disease course compared with nature course.

Improvement were also seen in shoulder ROM at one week to one month, and the period of total pain relief was at a mean time of 3.7 to 3.8 weeks. There were higher ASES Shoulder score in group B than in group C (P = 0.02) and higher DASH score in diabetic group in short term follow-up.

**Conclusions:**

Arthroscopic release provides effective and rapid improvements to shoulder motion and function, unrelated to the timing of surgery, in patients with frozen shoulder. The diabetic patients do not have functional outcomes as good as the nondiabetic patient at short-term follow-up.

## Introduction

Frozen shoulder, is commonly encountered in orthopedic practice. It affects 2%–5% in the general population [1,2], with more prevalence in women between the ages of 40 and 60 years [3, 4, 5]. It is usually characterized by decreased intra-articular volume and capsular compliance, which subsequently induce pain and cause limited range of motion [6].It is generally accepted that there are three phases during the natural course: freezing phase, frozen phase, and thawing phase. Each of these phases may last several months, and it usually takes 1 to 4 years for functional recovery [7,8]. Frozen shoulder commonly occurs in patients with certain comorbidities, the best known of which is diabetes. Several studies reported a high prevalence in patient with diabetes, ranging from 10~40% [9,10]. Generally, diabetic frozen shoulder cases were found to be more persistent and difficult to treat than nondiabetic frozen shoulder [11].

Treatment of frozen shoulder is controversial. The initial treatment includes a series of conservative therapies depending on the phase (i.e. physiotherapy, nonsteroidal anti-inflammatory drugs, corticosteroid injections…etc.) [7], followed by more invasive procedures in refractory patients. Surgical interventions includes open release, arthroscopic release, manipulation under anesthesia (MUA), or combination therapy [12]. Numerous studies have supported the role of arthroscopic capsular release for recalcitrant frozen shoulder [13]. However, few studies have focused on the optimal timing of surgical intervention and the surgical outcomes in diabetic patients.

The purpose of this study was to compare the effect of timing of arthroscopic capsular release and to compare the clinical outcomes between diabetic and nondiabetic patients in short-term follow up. We hypothesized that: 1. the patients with early surgical intervention can shorten the nature course of frozen shoulder with better outcomes, 2. the patients with diabetic frozen shoulder would have poorer clinical outcomes than nondiabetic patients.

## Materials and methods

All procedures performed in studies involving human participants were in accordance with the ethical standards of the institutional research committee of Kaohsiung Medical University Chung-Ho Mentorial Hospital. The IRB Number of this research is KMUH-IRB-2014079.We performed a retrospective review of a single surgeon, single institution, and consecutive series of patients over a period from January 2010 to December 2013. A total of 127 patients with primary frozen shoulder in the frozen phase were included, and thirty-two were diabetic. The mean age at the time of surgery was 55.6 years (± 8. 2 y, range: 39 to 72 years). These patients underwent arthroscopic capsular release and manipulation under anesthesia after conservative treatment was not successful. The inclusion criteria were: (1) difficulty using the affected arm in daily activities; (2) intolerable pain, especially night pain; (3) recurrent symptoms despite conservative treatment; and (4) pain more than 8 weeks. The exclusion criteria were (1) posttraumatic (i.e. fracture) or other systemic disease (i.e. rheumatic arthritis) causing secondary frozen shoulder; (2) unwillingness to undergo the operation or inability to receive follow-up; and (3) being unfit for anesthesia.

Demographic details included age, gender, diabetes, and the duration from symptoms onset to surgery. The duration of symptoms was recorded at the time of consultation, and the surgery was scheduled within two weeks. Patients were divided into 4 groups (Group A, B, C, and D) based on the duration at the time of surgery. The duration of symptoms were within 3 months, more than 3 months but within 6 months, more than 6 months but within 12 months, and more than 12 months in group A, B, C, and D, respectively.

### Surgical technique

Once fully anesthetized in the operation room, the patient was put in the lateral decubitus position and sterilized the entire upper arm. We assessed and documented shoulder ROM before the surgery. It usually had difficulty to insert the arthroscope into a shoulder joint due to capsular contracture, so a 19-gauge needle was inserted from the posterior arthroscopic portal. Saline solution was then introduced into the shoulder space in order to distend the contracted intra-articular space and observe the rebound. A initial inspection is performed to identify the long head of the biceps (LHB), areas of synovitis and contracted tissue, and possible coincidence pathology such as biceps tendon pannus. The scar and contracted tissue were released either by using a motorized shaver or radiofrequency device. We performed the sequential capsular release, including the superior capsule, coracohumeral ligament up to the base of coracoid process, the anterior capsule, and the inferior capsule. The posterior capsule was released through anterior viewing and working portals. Lastly, the subacromial space was checked to see if there was any rotator cuff tear or inflammatory change of bursa. Before the arthroscopic instruments were removed, a bolus of 15 to 20 mL of a combination of 0.5% bupimarcaine and 40mg triamcinolone acetate (Shincort) were injected into the glenohumeral joint. Subsequently, shoulder manipulation was performed. At first, the upper arm was hold and elevated forward in the sagittal plane to the maximum possible extent. Secondly, the arm was externally rotated in 0 degree of abduction, followed by gradual increase of abduction to 90 degrees. Finally, the arm was put in cross-chest adduction and internal rotation in 90 degree of abduction [14].

The postoperative rehabilitation protocol include sling protection, cryotherapy, supervised physiotherapy program including pendulum exercise and passive shoulder ROM immediately after the surgery. Patients were educated to perform home exercise program and received regular follow-up at orthopedic clinic.

### Assessment of clinical outcomes

Postoperative clinical outcome measurements were acquired at our clinic. Shoulder ROM was assessed pre- and postoperatively in three directions: forward flexion, abduction, and external rotation. The clinical outcomes were evaluated by the Disabilities of the Arm, Shoulder, and Hand (DASH) score [15], the American Shoulder and Elbow Surgeons (ASES) Shoulder score [15], the period of pain relief (defined as the time interval from receiving surgery to free from symptoms), overall duration of disease (defined as the time interval from onset of symptoms to free from symptoms), and satisfaction (range from 1 to 10, 1 means least satisfied and 10 means most satisfied).

### Statistical analysis

We used count (percentage) for describing distributions of categorical variables between groups, as well as mean or median (range) for continuous variables. Chi-square test was used for comparison of the different distribution between categorical variables and groups. To determine the differences of shoulder ROM, ASES score, DASH score, and the period of pain relief between groups with various timing of surgical intervention, two-way ANOVA test was used. In order to evaluate the effect of diabetes on the shoulder ROM, ASES score, DASH score, and the period of pain relief, we use independent t-test to determine the difference between diabetes and non-diabetes groups. *P <0* .*05* was considered statistical significance. All analyses were performed by SPSS for Windows software (version 19)

## Results

Between January 2010 and December 2013, 127 consecutive patients with diabetic and nondiabetic frozen shoulder were included in our study. Mean follow-up time was 29.9 months. There was no difference in age, sex, or diabetes distribution between the four groups (Table 1). The mean follow-up was longer in group D than in group A (P = 0.01) and group B (0.009).

**Table 1:** Demographics of patients.

	Group A	Group B	Group C	Group D	Total	*P* value
Mean age (year)	57.5±10.0	53.3±6.8	56.9±8.4	56.7±7.9	55.6±8.2	0.11
Gender (male/female)	7(29.2%) / 17(70.8%)	22(46.8%) / 25(53.2%)	5(19.2%) / 21(80.8%)	12(40.0%) / 18(60.0%)	46(36.2%) / 81(63.8%)	0.10
Diabetes	7/24 (29.2%)	9/47(19.1%)	6/26(34.6%)	10/30(33.3%)	32/127(25.2%)	0.53

### Range of motion

Mean abduction, forward flexion, and external rotation improved significantly after surgery in each group (P < 0.0001). However, there was no statistical difference in the pre-operative and post-operative shoulder ROM between the four groups and between diabetic and nondiabetic groups (Table 2).

**Table 2:** Outcomes of surgery (group A to D, nondiabetic and diabetic).

		Group A	Group B	Group C	Group D	Total	P value^a^	P value^b^
**Follow-up** (month)		27.2 (±10.1)	28.2 (±10.3)	28.9 (±9.8)	35.7 (±10.1)	29.9±10.5		0.005**
**Forward flexion** [degree (range)]								
Preop	Non-DM		92.8 (±28.7)	93.8 (±23.5)	79.0 (±18.5)	89.2 (±24.0)	0.09	0.28
	DM	85.7 (±12.7)	102.2 (±25.9)	91.7 (±22.3)	101.0 (±27.7)	96.3 (±23.5)		
Postop	Non-DM	164.1 (±24.0)	163.0 (±21.6)	163.5 (±20.6)	168.0 (±19.8)	164.4 (±21.2)	0.40	0.56
	DM	154.3 (±20.7)	163.3 (±21.8)	155.0 (±22.6)	168.0 (±15.5)	161.3 (±19.8)		
**Abduction** [degree (range)]								
Preop	Non-DM	82.9 (±18.3)	84.5 (±24.9)	81.3 (±25.3)	85.0 (±18.2)	83.6 (±22.4)	0.57	0.99
	DM	87.9 (±16.3)	83.3 (±32.0)	90.0 (±22.8)	86.0 (±20.7)	86.4 (±23.0)		
Postop	Non-DM	174.7 (±10.5)	168.2 (±22.9)	165.0 (±16.9)	170.3 (±10.1)	169.1 (±17.7)	0.81	0.26
	DM	171.4 (±14.6)	168.3 (±14.6)	165.0 (±25.1)	175.5 (±10.1)	170.6 (±15.6)		
**External rotation** [degree (range)]								
Preop	Non-DM	20.0 (±6.6)	18.3 (±9.8)	26.3 (±13.5)	16.8 (±8.5)	20.0 (±10.3)	0.94	0.07
	DM	17.1 (±9.5)	22.2 (±11.2)	18.3 (±6.1)	19.5 (±10.9)	19.5 (±9.7)		
Postop	Non-DM	39.4 (±7.1)	39.3 (±7.5)	36.5 (±10.0)	38.3 (±7.7)	38.5 (±8.0)	0.09	0.37
	DM	32.9 (±13.5)	37.2 (±7.6)	31.7 (±9.8)	38.5 (±7.1)	35.6 (±9.4)		
**DASH** [mean (range)]								
Non-DM		7.4(±7.7)	7.0(±7.6)	12.2(±13.7)	8.2(±11.4)	8.4 (±10.1)	0.04	0.08
DM		14.3(±11.1)	8.1(±9.9)	20.4(±21.7)	12.0(±7.0)	13.5 (±12.5)		
**ASES** [mean (range)]								
Non-DM		88.3(±12.7)	89.7(±10.5)	82.2(±18.7)	86.6(±11.1)	87.2 (±13.2)	0.14	0.03
DM		79.3(±14.3)	93.0(±7.7)	73.1(±30.3)	81.2(±11.3)	82.6 (±17.1)		
**Pain relief** [weeks (range)]								
Non-DM		3.4 (±0.7)	3.7 (±0.5)	4.4 (±0.6)	3.3 (±0.6)	3.7 (±3.0)	0.88	0.81
DM		5.1 (±1.1)	2.4 (±1.0)	3.0 (±1.2)	3.9 (±0.9)	3.6 (±2.4)		
**Duration of symptoms** (month)								
		2.7 (±0.5)	5.6 (±0.7)	11.2 (±1.7)	38.4 (±23.0)	14.0 (±17.8)		<0.0001**
**Duration of disease** [weeks (range)]								
Non-DM		14.2 (±2.3)	26.2 (±2.9)	49.4 (±8.5)	151.9 (±76.8)	55.4 (±7.3)	0.89	<0.0001**
DM		15.4 (±4.4)	25.1 (±2.9)	48.0 (±8.4)	157.5 (±127.1)	68.7 (±12.5)		
**Satisfaction** [mean(range)]								
Non-DM		7.5 (±1.9)	8.1 (±1.0)	7.7 (±1.2)	8.1 (±1.2)	7.9 (±1.3)	0.90	0.13
DM		7.0 (±1.9)	8.2 (±1.0)	8.2 (±1.9)	7.8 (±1.2)	7.8 (±1.5)		

P value^a^: between non-DM and DM

P value^b^: between four groups

DASH, Disabilities of the Arm, Shoulder, and Hand score; ASES, American Shoulder and Elbow Surgeons score

** Values are statistically significant (P < 0.05)

### Functional scores and satisfaction

The clinical outcome can be associated with functional outcomes, time for free of pain, and pain severity. There were higher ASES Shoulder score in group B than in group C (P = 0.02), and higher DASH score in diabetic group in short term follow-up. The time interval from the surgery to free from symptoms was 3.6 weeks for nondiabetic and 3.7 weeks for diabetic, which showed no statistical difference. Overall duration of disease showed great difference between four groups (P<0.0001), with increasing time from group A to group D, but no difference between diabetic and nondiabetic groups

### Complications

In this series, there was no intraoperative or postoperative complications including fractures, dislocations, symptoms of acute rotator cuff tear or biceps tendon injury, axillary nerve dysfunction, or infection.

## Discussion

In current study, the etiology of frozen shoulder is not clearly understood. Patients usually suffered from shoulder pain initially, accompanied with limited shoulder range of motion at a later time. Some authors claim that the nature course may take 2–7 years. Hand et al. [16] reported 40% of patients with untreated frozen shoulder have prolonged symptoms. Its long-standing nature course for resolution may affect our quality of life. Thus, we consider if early surgical intervention may shorten the nature course of frozen shoulder. By reducing pain and improving shoulder function after surgery in short period of time, patient can soon get back to work and have high quality of life. After searching online database, little literature has emphasized the effect of surgical timing on the clinical outcomes. Our goal is to evaluate the effect of timing of arthroscopic surgery and to know if overall duration of frozen shoulder can be shortened.

Arthroscopic capsular release has proven a successful procedure for the treatment of frozen shoulder [17, 18]. Baums et al. [19] and Smith et al. [20] had demonstrated considerable early improvement in range of motion, pain, and overall shoulder function after arthroscopic capsular release. The clinical relevance of our findings were similar in our study. There was a significant and rapid improvement after arthroscopic capsular release, unrelated to the timing of presentation, in these patients. With a great improvement in shoulder ROM at a mean follow-up of 29.9 months and no surgical complications, we concluded that this kind of treatment is clearly beneficial. The overall duration showed great different with increasing time from group A to D (Table 2). Despite diabetic or not, patients in group A, B, and C demonstrated much shorter disease course, ranging from 14.2 to 49.4 weeks, compared with spontaneous recovery. We found the mean overall duration showed more than 150 weeks (2.9 years) in group D. Comparing with other groups of patients, it was quite longer. We postulated it related to prolonged time interval from symptom onset to surgery, which usually lasts for more than 24 months. At one to three days after arthroscopic capsular release, most patients reported great pain relief and less frequency of night pain. Improvement were also seen in shoulder ROM at one week to one month and the period of total pain relief at a mean time of 3.7 to 3.8 weeks (Table 2). We propose this finding as an indication for early surgical intervention in preference to conservative treatment. One special finding in our study was the higher ASES Shoulder score in group B than in group C. This data may provide the information to explain better recovery pattern in patients who receive surgery early.

To date, little studies focused on the role of arthroscopic surgery in diabetic frozen shoulder. The efficacy of operative management in patient with diabetes remains unclear. Cho et al. [9] compared clinical outcomes after arthroscopic treatment in diabetic and nondiabetic patients with refractory frozen shoulder. The shoulder range of motion and mean ASES score were worse in the diabetes group in the early postoperative follow-up. Mehta et al. [10] compared the clinical outcomes in diabetes and nondiabetic patients after arthroscopic capsular release in a prospective study. The clinical score was significant lower in diabetic group 6 months postoperatively. In brief, most present studies have reported that patients with diabetes tend to have worse outcomes in short-term or mid-term follow-up but have similar long-term outcomes. The results were consistent in our study. Despite no statistical difference regarding improvement of shoulder ROM, the period of pain relief, overall duration of disease, and satisfaction between diabetic and nondiabetic group. There was significantly higher DASH scores (13.5 vs. 8.4, P = 0.04) at short-term follow up. Thus, the final outcome of arthroscopic release in patient with diabetes may not as good as in patient with nondiabetes.

The strengths of our study is that we had included large number series of patients with strict inclusion and exclusion criteria. All the patient receives the operation with same approach and technique by the single surgeon.

This study has some limitations. The most prominent limitation was the lack of a control group. Including a control group may have enabled comparison with the natural progression of recovery. Moreover, we lacked preoperative functional scores (ASES and DASH) for comparing the level of improvement after surgery.

## Conclusions

Arthroscopic release provides effective and rapid improvements to shoulder motion and function, unrelated to the timing of surgery, in patients with frozen shoulder. The diabetic patients do not have functional outcomes as good as the nondiabetic patient at short-term follow-up.

## Supporting information

S1 File Frozen shoulder (patient data-new). (XLSX)
